# Embodied word learning in schools and sustained attention in virtual reality

**DOI:** 10.1038/s41539-025-00395-2

**Published:** 2026-01-10

**Authors:** Maja Rudling, Theodor Rumetshofer, Jens Nirme, Johan Mårtensson

**Affiliations:** 1 Lund University, Department of Clinical sciences, Lund, Sweden; 2 Lund University Cognitive Science, Department of Philosophy, Lund, Sweden

**Keywords:** Mathematics and computing, Neuroscience, Psychology, Psychology

## Abstract

This study examined whether immersive virtual reality (VR) supports second-language vocabulary learning in school-aged children compared to passive computer-based training. Seventy-three Swedish middle-school students learned novel words by assembling objects in VR and by viewing object assembly on a computer screen in a crossover design. Word recall was tested after each condition, and individual differences in language aptitude and sustained attention were assessed. Overall, recall was higher after computer-based training than VR. However, sustained attention was the strongest predictor of learning outcomes, and the difference between conditions was (marginally) significant only for children with higher sustained attention. No VR behavioural measures (gaze, assembly time, rotation) explained learning outcomes. These findings suggest that VR may not universally enhance vocabulary learning but could benefit learners with specific attentional profiles. Further research should explore how VR design and training duration influence language acquisition in school settings.

## Introduction

Small children learn their native languages (L1) through a complex interplay of social, cognitive and explorative behaviours, where their active use of their bodies facilitates the association between objects and the corresponding words^1^. When older children and adults learn new languages (L2) in school settings, they are seldomly afforded the same approach. Instead, vocabulary in new languages is often taught by linking the new L2 word with the corresponding L1 word, in a process of transfer that is sometimes called *parasitic* word learning^2^. According to the unified competition model of second language learning^3^, the anchoring of the semantic meaning in parasitic L2 word learning is not as robust as it is for L1 learning, and the word-object associations in traditional L2 language learning is therefore also weaker^4,5^. To be able to build a strong vocabulary in an L2, the learner has to establish new conceptual and semantic representations for the words that are at least partly independent from the L1 equivalents^3^. As there is a large group of L2 learners who struggle with second language learning, and who may benefit from alternative learning methods^6^, this pinpoints the need for well-thought-out pedagogical approaches for L2 learning. One potential way to help L2 learners establish a stronger conceptual representation to words could be to make L2 learning more similar to L1 learning.

A possible key to how L1 and L2 learning differs from each other is the exploration that goes into first language learning. The *grounded cognition* perspective proposes that learning and thinking cannot be separated from the body that performs these processes, nor from the environment and context in which it is performed^7^. From this viewpoint, language use and language learning are both embodied, ingrained in sensorimotor experience, and situated in a learning context^8,9^. This means that language learning is both experiential and multimodal. The experiential aspect, meaning *learning by doing*, is typically present in L1 learning, where the infant picks up, tastes, feels, and experiments with the objects that they learn the words for. Multimodal learning means that we utilize several sources of information in the learning process: our hands, eyes, ears, etc. The resulting integration of motor action, sensory perception, and cognitive processes has been shown to lead to enhanced cognitive processing and deeper learning, also of language^10–12^. However, this kind of situated learning requires a meaningful integration of the activity and the linguistic content, ensuring that the language element (e.g., the name of an object) is embedded in the task itself rather than detached from it^9^. Without this alignment, the learning scenario risks becoming artificial and less engaging. From a theoretical perspective, if L1 learning is indeed more robust than L2 learning, it could be beneficial to make L2 learning more alike to L1 learning by making it more experiential and multimodal. This means encouraging L2 learners to use multiple senses and their body to explore their surrounding when learning new words. However, whether such an approach is beneficial in real life school settings is still an open question. As the world has become a more globalized and interdependent place, where we encounter more and more languages in our everyday life, L2 proficiency is also increasingly important. Finding ways to support second language learning in schools is therefore a worthwhile endeavour.

Virtual reality (VR) with a head-mounted display enables users to experience being in another environment by using both sight and hearing, and makes it possible to affect the virtual environment by their actions. A virtual environment is a three-dimensional simulation that responds to your movement, in which you can explore and manipulate the world by using hand controls, making it more immersive than passively viewing of 3D worlds^13^. Researchers in the field of learning technology and educators alike have highlighted the potential for VR as a tool for dynamic and immersive learning. Suggested benefits include increased embodied learning and learner motivation as well as reduced distractions, and stronger links between what is taught and the real-world applications through situated learning in naturalistic learning settings^14–18^. Hence, it is easy to see that VR has strong potential as a tool to make L2 language learning emulate L1 acquisition. So far, however, the research on VR in education is still limited and varies highly in study design, measured outcomes, learning material, and learning environments. For example, there is no clear agreement on how to define VR, as the term can include everything from three-dimensional videos shown on a computer screen to the significantly more immersive head-mounted VR systems^19–21^. This makes it difficult to compare results between studies and hinders the process of identifying which mechanisms are behind the potential benefits of learning in VR. As of yet, a small number of studies has focused specifically on VR in language education, and this research field is likewise varied; which ages, which teaching method, and which aspects of language and communication are studied differs from study to study^20,21^. In several studies, VR is shown to have potential as a tool for language learning, being beneficial both for learning and for learner’s motivation^14,20–22^. For example, Legault and colleagues found that young adults increased their foreign language vocabulary more when they were using head mounted VR to interact with a virtual environment than when they were learning to associate the written English word with their mandarin counterpart on a computer screen^23^. However, the results of experimental studies on the effectiveness of VR learning over other types of teaching methods has not been conclusive, with even potential indications of a more positive effect of the control condition over the VR training^24,25^. This is true also for VR language learning. For example, while Cheng and colleagues found that the university students who participated in their study did improve their cultural understanding and reported that they enjoyed playing the VR game, the vocabulary improvement was larger in the PC comparison condition than in the VR condition^26^. To this day, the majority of research on language learning in VR has been focused on foreign language learning in university students, and much less focus has been on the effectiveness of VR in children^20,27^. Indeed, according to a recent systematic review, there is a distinct lack of research on the applicability and effectiveness of VR language learning in children, on studies that focus on lexicon and vocabulary learning, and on studies that systematically test aspects of VR learning with controlled experiments^20^. This conclusion is supported by another recent systematic review that specifically highlights the need for more studies comparing VR to more conventional teaching methods for children^21^. With VR equipment becoming more affordable and therefore increasingly feasible to use in schools for children, research on the suitability, challenges, and benefits of VR in language teaching have also become more urgent in order to support policy making^27^.

If we are to evaluate the implementation and effectiveness of VR language learning in schools, we must take learner individual differences into account^28^. It is unlikely that one VR language learning approach will fit all learners equally well. Instead, individual differences in terms of interest in VR and games, motivation, as well as learning strategies and behaviour has been suggested as potentially important factors affecting how well a VR learning tool may work^20,29^. Furthermore, cognitive aspects such as executive functioning, working memory, and general language ability are likely to influence VR language learning^18,23,30,31^. In a study comparing learning in an interactive learning environment on a computer screen to a regular picture-word-association task, learners’ working memory ability predicted vocabulary acquisition in the interactive setting but not in the passive comparison condition^30^. However, when using immersive head-mounted VR in a follow-up study, the same research group found no correlation between working memory and word learning in either learning condition. Instead, they found that L1 proficiency, measured as L1 vocabulary, was predictive of L2 vocabulary attainment^23^. Aspects of executive functioning such as being able to inhibit automatic responses have also been found to be linked to L2 learning proficiency^32–34^. A possible explanation for this association is that L2 users need to actively inhibit their more automatic L1 responses to be able to learn and use their second language without interference^31,32^. However, inhibition has not yet been extensively studied in relation to learning in virtual reality. Further, to our knowledge, the link between VR language learning and sustained attention, which is an executive function strongly linked to inhibition, has not previously been investigated in the research field. Sustained attention is the ability to continue focusing on a task over an extended period of time and to not let the mind wander. It is often measured with the Sustained Attention to Response Task, or SART^35^, in which participants are asked to push a button as quickly as possible after a digit appears on the screen, except for when that digit is a 3. At first glance this task seems easy, but with many repetitions over several minutes it puts high demands on the ability to inhibit the automatic response to push the button when anything appears on the screen, as well as the ability to sustain attention and stay vigilant despite it being a repetitive and boring task. How well one performs this task has been linked to attention in the everyday life^35,36^ and it has been found useful for identifying children with Attention Deficit Hyperactivity Disorder/ADHD, a condition that is largely defined by executive dysfunction^37,38^.

The goal of this study was to investigate the effect of active, embodied learning on word-object associations in a school setting. To do this, we made an experimental setup where school-aged children learned words in an interactive and immersive VR environment using head-mounted displays and compared it to an associative word learning task on a computer display (PC). To make the learning set-up resemble infant, non-parasitic L1 learning, we used the words and objects of *the ancient farming equipment paradigm*^39^. This paradigm utilizes old Finnish agricultural tools which possess both unknown names and unfamiliar shapes to the participants, making them essentially phonotactically sound non-words and “non-objects”. Unlike naming familiar objects in a novel language through parasitic language learning, this paradigm necessitates that newly learned objects are named through a direct visual-phonological link. This enables us to both mimic L1 learning and ensure similar baseline knowledge between participants at the same time. In the VR environment, the children used their hands to assemble objects from pieces and were then presented with the name of the object both in text and auditory. To our knowledge, this is the first VR learning study that systematically investigates the effect of using one’s hands to manipulate and assemble objects on vocabulary learning, as previous studies have mainly allowed participants to view or lift up and rotate objects in the VR environments^21^. The specific aims of this study were threefold: Firstly, we wanted to know if there is an advantage of interactive VR training over more traditional associative word learning on a PC screen. Secondly, we wanted to know if there are individual differences in terms of general language ability or executive functioning that factor into the relative usefulness of VR versus PC for word learning. Thirdly, we wanted to explore learners’ behaviours in the VR environment that were associated with learning, such as gaze patterns, hand movements, and how long they spent on the task.

## Results

Analyses focused on PC and VR Recall scores as the primary outcome measures, calculated as the number of correctly identified words per training block (maximum = 18). Predictor variables included sustained attention, assessed with the Sustained Attention to Response Task (SART; accuracy on no-go trials, range 0–100%), and language aptitude, measured with LLAMA B (associative learning) and LLAMA D (auditory implicit learning). Additional VR-specific behavioural measures were Word Gaze (percentage of observation phase spent looking at the written word), Assembly Time (seconds to complete object assembly), and Rotation (cumulative angular rotation during observation). Details of task implementation and scoring procedures are provided in the Methods section. Descriptive statistics are reported in Table 1.

**Table 1:** Descriptive statistics

	*N*	Mean	SD	Minimum	Maximum	Skewness	Kurtosis
**PC Recall**	72	5.28	1.54	2.50	10.67	0.92	1.53
**VR Recall**	71	4.81	1.34	2.67	9.00	0.77	0.77
**SART (%)**	73	27.01	19.27	0.00	88.00	1.17	1.18
**Llama B**	73	4.19	2.77	0	14	0.81	1.26
**Llama D**	72	6.86	4.04	0	16	-0.02	-0.87
**Word Gaze (%)**	72	19.44	8.90	6.03	53.69	1.61	3.15
**Assembly time**	64	28.49	10.76	15.49	64.06	1.42	2.17
**Rotation**	64	1444.67	477.68	512.77	2450.85	0.32	−0.82
**Age (months)**	73	139.01	12.86	122.00	157.00	0.11	−1.76
**SES**	72	3.06	0.77	1.50	4.00	−0.12	−1.21

### Correlational analyses

First, we performed correlation analyses to investigate the relation between the VR and PC recall scores, and the SART and Llama scores, and the potential control measures (age, SES, gender, school, bilingualism, how often the child plays computer games, and how often the child plays in VR). The VR and PC recall scores correlated significantly with each other (*r* = 0.25, *p* = 0.039, *95%* ^*BCa*^*CI* = -0.07; 0.47, *n* = 70). The only other variable that significantly correlated with the recall scores was sustained attention, measured by the SART score, which was positively correlated with the PC recall score (*r* = 0.38, *p* = 0.001, *95%* ^*BCa*^*CI* = 0.14; 0.59, *n* = 72) but not the VR recall score (*r* = 0.17, *p* = 0.150, *95%* ^*BCa*^*CI* = -0.08; 0.44, *n* = 71). Neither LLAMA B (associative learning) nor LLAMA D (auditory implicit learning) scores, nor any of the potential control variables correlated significantly with the PC or VR recall scores (Supplementary Table 1) and were therefore not included in the further analyses of the recall scores.

### Learning conditions and sustained attention

**Fig. 1:**
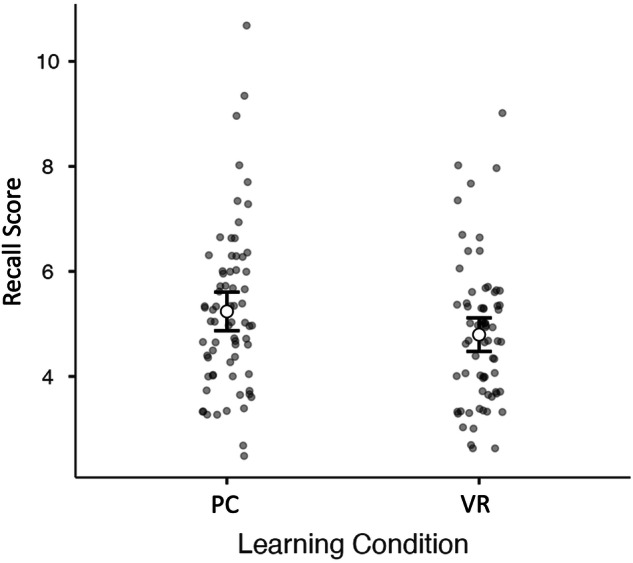
The recall scores across learning conditions. Estimated marginal means (white circle) and 95% confidence intervals (error bars) of the Recall Scores between the two learning conditions (PC and VR). Gray dots represent individual data points.

To test the effect of learning conditions on the recall score, we performed repeated measures ANOVA analyses, where the PC and VR recall scores were entered into the model first. There was a significant effect of learning conditions on the recall score (*F(1,69)* = 4.36, *p* = 0.041, partial η^*2*^ = 0.06), where the PC learning condition gave significantly higher recall scores (mean difference 0.44, SE = 0.21; Fig. 1). We then added the SART score into the model as a continuous variable. The main effect of the SART score on word recall was significant (*F(1,68)* = 9.87, *p* = 0.002, partial η^*2*^ = 0.13), while the interaction between the learning condition (PC or VR) and the SART score was not (*F(1,68)* = 2.38, *p* = 0.128, partial η^*2*^ = 0.03). When SART was added to the model, the main effect of the learning condition was no longer significant (*F(1,68)* < 0.01, *p* = 0.972, partial η^*2*^ = 0.00), indicating that the SART score may be a more important factor in explaining variation in recall scores. To explore this result further we split the dataset based on the median of the SART score (0.24) and performed the repeated measures ANOVA on the two median split groups separately. The results showed that for the below-median group there was no significant difference between the PC and VR recall scores (*F(1,37)* = 0.73, *p* = 0.399, partial η^*2*^ = 0.02), whereas for the above-median group, the difference was marginally significant despite the decreased statistical power of these analyses (*F(1,31)* = 4.03, *p* = 0.054, partial η^*2*^ = 0.11). This could indicate that it is only for the children with higher sustained attention that the PC recall score was higher than the VR recall score, whereas for the children with lower ability to sustain attention in repetitive tasks the VR and PC conditions were equal. The low and high SART score groups also differed significantly in terms of overall word recall (VR and PC combined; *t*(71) = 2.31, *p* = 0.024, Cohen’s d = 0.54).

### VR behavioural measures

To test if VR-specific behaviours predicted word learning, we examined *Word gaze*, *Assembly time*, and *Rotation*. We first correlated Word gaze with the VR recall scores, but found no significant correlation (*r* = −0.06, *p* = 0.609, ^*BCa*^*CI 95% CI* = −0.27; 0.14, *n* = 71). Further, Word gaze was not correlated with the SART score (*r* = 0.08, *p* = 0.510, *95%* ^*BCa*^*CI* = −0.11; 0.26, *n* = 72) nor SES (*r* = −0.07, *p* = 0.583, *95%* ^*BCa*^*CI* = −0.31; 0.18, *n* = 71). Word gaze was, however, significantly correlated with age (*r* = 0.34, *p* = 0.003, *95%* ^*BCa*^*CI* = 0.13; 0.53, *n* = 72) as older children seemed to have looked longer at the written word. Additionally, there was a significant difference between genders (t(70) = 3.35, *p* = 0.001, *Cohen’s d* = 0.79) where girls (*M* = 0.23% of the time the word was displayed, *SD* = 0.10) looked longer than boys (*M* = 0.16%, *SD* = 0.06). Next, we correlated Assembly time and Rotation with the VR recall scores, SART, and age and gender, as these two control variables were related to word gaze behaviour. Assembly time did not correlate significantly with the VR recall score (*r* = -0.01, *p* = 0.954, *95%* ^*BCa*^*CI* = −0.25; 0.20, *n* = 63) or the SART score (*r* = 0.02, *p* = 0.897, *95%* ^*BCa*^*CI* = −0.22; 0.26, *n* = 63). It was negatively correlated with age (*r* = −0.26, *p* = 0.038, *95%* ^*BCa*^*CI* = −0.45; −0.04, *n* = 63), as younger participants took longer to assemble the objects, but not significantly correlated with gender (*r* = −0.04, *p* = 0.779, *95%* ^*BCa*^*CI* = -0.29; 0.23, *n* = 63). The amount of rotation during the observation phase also did not significantly correlate with the VR recall score (*r* = −0.01, *p* = 0.914, *95%* ^*BCa*^*CI* = -0.25; 0.25, *n* = 63). It was, however, marginally correlated to the SART score (*r* = −0.24, *p* = 0.056, *95%* ^*BCa*^*CI* = −0.44; −0.01, *n* = 63). Further, it both significantly correlated with age (*r* = −0.37, *p* = 0.003, *95%* ^*BCa*^*CI* = −0.56; −0.11, *n* = 63) and gender (*r* = −0.38, *p* = 0.002, *95%* ^*BCa*^*CI* = −0.57; −0.15, *n* = 63). Younger participants tended to rotate objects more than older participants, and the same was true for boys (*M* = 1638.86; *SD* = 458.39) compared to girls (*M* = 1273.33, *SD* = 431.77). While the eye tracking measure Word gaze correlated with rotation time (*r* = −0.35, *p* = 0.005, *95%* ^*BCa*^*CI* = −0.55; −0.09, *n* = 64), it did not correlate with time to assemble (*r* = −0.13, *p* = 0.325, *95%* ^*BCa*^*CI* = −0.32; −0.11, *n* = 64).

## Discussion

The first aim of this study was to investigate if children learn novel words better when they train in a high-immersive environment, assembling objects using their hands in VR, or if learning the same object-words combinations using a passive, regular low-immersive PC program where children passively view the construction of the objects is just as effective. Our results point to a slight, potential advantage of using a PC for word learning rather than the more involved VR learning. At first glance, our results look to go against previous research in the field of VR for language learning, as many of those studies showed an advantage for VR^14,20–22^. However, previous research in this field is still limited in its scope and the results have not been conclusive^20,24,25^. Further, to our knowledge, the current study is the first experimental study where VR learning has been compared to equivalent PC learning in a school setting.

A potential explanation for the ambiguity of previous research on VR as a learning tool, as well as a factor to why VR might not always be superior to alternative learning methods, is that studies differ in the type of knowledge that learners are expected to obtain. According to Anderson and Krathwohl’s taxonomy of knowledge, factual knowledge pertains to knowledge of terminology and facts whereas procedural knowledge pertains to knowledge of skills, techniques, procedures, and methods^40^. There have been indications that VR might lend itself better to obtaining procedural knowledge than factual knowledge^18^. For example, in the study by Cheng et al.^26^, participants who practiced in VR learnt how to bow correctly according to Japanese culture, something that is considered procedural knowledge. However, they did not learn Japanese vocabulary despite having opportunities to do so in the virtual learning environment, which could arguably be categorized as factual knowledge. Correspondingly, in our study we found no advantage in vocabulary learning in the VR environment, but it is possible that if we instead had tested how well the participant’s learnt how to assemble the objects, we would have gotten a different result as this would entail procedural learning instead of factual. Assuming that VR does indeed facilitate procedural learning, and that participants in the VR condition were primarily engaged in such learning, this may have diverted cognitive resources from word acquisition, potentially explaining the comparatively lower performance in VR learning observed in our study.

We do not find that the relative advantage of PC over VR in our study necessarily contradicts the notion that embodied and situated learning might be beneficial for obtaining a second language. In the embodied perspective, infants don’t just learn new words by associating a word to an object or a concept. By using their bodies to explore the world around them they *construct meaning*^8^. In our current study, we don’t allow participants to go this extra step since they never move beyond the form of the object and into the semantic meaning of it. Relatedly, situated language learning presupposes that the learning activity and the linguistic component are meaningfully integrated^9^. In our design, however, the target words were introduced only after the object was completed, which contradicts this principle and may have limited the learning potential of the VR task. If the VR learning task in this study instead had been meaningful for the usage of the objects and the word learning aspect had been embedded into the object assembling task, it is possible that this would have facilitated a deeper association between the objects and words. One way of doing this could be to place participants in a virtual old Finnish farm, having a character guide the child through a goal-directed task (‘We are going to make a *riitmott*, can you help me find the parts?’), providing them corrective feedback (‘No, this piece is for an *auma*, can you find the piece for a *riitmott*?”), and allowing them to use the objects for what they are intended for after assembling them. Beyond emulating infant embodied and situated language learning more closely, this would also allow learners to deepen their semantic understanding of the words. Another important process of infant language acquisition is social learning, and for L2 learning to resemble L1 learning this aspect should ideally be incorporated^41^. By including a social agent that is guiding the learner and providing feedback, this aspect would also be simulated in the VR-task. Lastly, and in relation to the previously mentioned different types of learning, this alternative VR setup could also allow participants to tap into procedural learning. All these factors may help scaffold the vocabulary learning. Further, extended and multimodal learning is by itself insufficient to explain how small children learn new languages so fast. For one, infants require many exposures to learn both word form and meaning and build this knowledge incrementally over an extended period of time^42,43^. In the current study, participants were only exposed to each word once during the learning phase and only trained for one week with each learning condition. Previous research has found that VR learning may take up to four training sessions before being effective for vocabulary learning^44^, and being in a VR environment in itself can be distracting, especially in the beginning. Therefore, repeated exposure to the words as well as longer training periods could potentially be beneficial for VR word learning. Such a set-up in an experimental study could also enable the study of whether VR training differentially affects encoding, consolidation, and long-term retention of novel words compared to non-interactive PC training.

The second aim of this study was to examine if individual differences in cognitive abilities like sustained attention and general language skills are linked to the effectiveness of VR as a learning tool for different children. Although the two included measures of general language ability were not correlated with the word recall scores in neither the VR or PC conditions, we did find that the ability to sustain attention to a boring, repetitive task was positively correlated with the PC learning condition and not the VR learning condition. Further, when the sustained attention measure was added to the PC vs. VR model, the learning condition was no longer a significant factor of word learning, but sustained attention was. This suggests that it may be an important factor for understanding who benefits from using VR as a learning tool. The reality of children’s school environment is that it is not always ideally suited for all learners. For one, the environment can often be noisy and full of distracting stimuli^45^. Our results indicate that children with better ability to sustain attention to repetitive tasks are the ones for which the PC training seem beneficial. In contrast, for the children who struggled to sustain attention, the PC and VR environments seem to elicit equal learning opportunities. The VR environment in the current study was deliberately designed to minimize visual distractions, and the learners were alone in a virtual environment without many details and no other activities available beyond the training task. These factors have previously been found to support learners’ ability to focus^46^. It is thus possible that this environment supports children’s concentration abilities and therefore is conducive for the children that struggle with sustained attention in their regular learning environment.

It has been proposed that VR puts high demands on working memory and learners’ limited processing abilities which thus leads to increased cognitive load^18^. In a study combining VR training with online EEG measurement, Makransky and colleagues found that the VR environment increased the cognitive processing of the student participants compared to the equivalent PC training. While participants reported feeling more present in the VR condition, they still learned less than in the PC condition^25^. The authors suggest that the higher levels of engagement the participant felt in turn increased the cognitive load. In tasks that are already cognitively demanding, such as learning can be, more engagement might thus actually be detrimental to the learning outcome. Although we neither measured cognitive load nor participants engagement in this study, we can make some possible inferences based on the differences of the two learning conditions and the link to the SART measure. Firstly, the task of assembling objects in the VR environment likely requires more cognitive resources than passive viewing of the same task on PC, simply based on it being an active task. Secondly, the VR environment, while designed to be devoid of distraction, is still richer than the very plain PC user interface, which might have added additional strain on attentional load during the task. Lastly, when asked by the test leaders and teachers, participating children generally reported that they found the VR task fun, but we got no similar remarks for the PC task. Arguably, the interactive VR task should have invited more active engagement than the passive watching task in the PC condition, and VR has previously been found to have a positive effect on user motivation^21^. Speculatively, if the VR assembly task required more cognitive resources than the PC equivalent, and if the VR task was more engaging to the participants, these two factors may have increased the demands on the children’s attentional resources, possibly contributing to the higher PC result in the overall analysis. Further, it is possible that this same increased engagement in combination with higher cognitive demands could explain the lack of difference between VR and PC in the low-sustained-attention group, if it is the case that children with lower ability to sustain attention actually benefit from the more active learning task, but that the increased cognitive load conceals this effect. If so, then the less interactive PC condition isn’t necessarily generally advantageous, but instead the way the learning tasks were constructed as well as the way we measure the outcomes may have artificially made it seem that way. While still hypothetical, if the cognitive load could be lessened while retaining the engagement in the task, the VR environment might even be beneficial over other types of training for this group of children. A goal for future studies is to test this by manipulating levels of cognitive load in relation to engagement in VR learning tasks, for example by shortening the training sessions, increasing the length of training periods, varying the amount of words the learners is exposed to, or by letting the learner set the pace of the training and number of repetitions^21^. Another aspect related to learner motivation is the previously mentioned disconnect between the object-assembling task and the exposure to the word, which is presented only after the assembling is finished. If the learners experienced the task as artificial as a consequence of this, it may have made the task lack in meaningfulness, and in turn decreased learner engagement. If so, increasing the situatedness of the task (for example through the changes of the VR task suggested earlier in the discussion) may be a way to increase engagement without increasing cognitive load, and thus potentially improving the VR environment as a language learning tool.

The third aim of this study focused on measures of learner behaviour in the VR environment and the potential link to learning. More specifically we looked at gaze behaviour towards the written name of the assembled objects, the time it took participants to assemble the objects, and how much they rotated the objects after assembling them. None of these measures significantly correlated with how well the participants learned the words. However, with these additional measures we were able to identify differences in VR behaviour which were associated with age (assembling time and object rotation), and gender and sustained attention (object rotation). As such, VR has a built-in potential for understanding the mechanisms of the learning process more in detail, which may prove useful to identify which learners benefit of VR training and how to design optimal VR environments. For example, in our study learners on average only watched the written word for around 2 seconds, indicating that the full 10 s that the word was displayed may be unnecessarily long. This observation could reflect the quickness of which proficient readers can decode a word, as well as the redundancy of having the word both written and spoken. The redundancy may in fact add extraneous cognitive processing demands from the splitting of the attention between identical information from two different sources, which in turn may take away resources better spent on the learning^25,47^. This is something to have in mind in designing learning environment as VR already can put higher cognitive demands on the learner^25^. Further, as younger and older children seemed to have approached the VR task slightly differently despite the quite small age range of this study, it might be wise to consider the age of the target learners when designing VR learning environments.

The current study has a number of limitations. First, although we did cover both sustained attention and two general language learning measures by including the SART and the two Llama tests, there are other measures of individual variability that would have been interesting to add to better understand which children benefit more or less from VR learning. For example, other measures of executive functioning as well as spatial ability, and measures of learner motivation, interest, and engagement could shed light on the processes behind successful learning in VR^28^. Second, in the current study we focus on word recall as our outcome measure and do not find any clear advantage of VR over PC, but as we have mentioned above, it is possible that the children learned other things in the VR environment that we have missed. It would therefore be interesting to measure the learning of the assembly task as well as more measures of language learning, such as long-term retention of the words, to further understand the potential scope of VR learning and what specific types of learning it is best suited for. Additionally, the recognition task used to assess word learning in the current study may not have been the most suitable. Other word learning assessment tasks, for example a naming task, may be more sensitive, and future studies should test whether that makes a difference in the relative advantage of VR versus PC. Third, although the design of the VR learning task was informed by creating a controlled experiment, as discussed previously, this may have made it less situated and naturalistic as a learning environment. This, in turn, may limit the ecological validity of the conclusions about the absolute usefulness of VR as a language learning tool. Fourth, one goal of this study was to realistically test the implementation of VR learning in Swedish schools. This meant training and testing children in their everyday life. However, this implementation in turn limited the control over external factors such as background noisiness, distractions, and other stressors. Although the number of participants included in the study is not insubstantial; to increase generalizability and compensate for the lack of control it would have been preferable to study more children in additional schools and with a wider range of ages and socioeconomic backgrounds.

The results of this study suggest that VR may not always be the ideal option for implementation in middle school language teaching. Indeed, in this study we found that in the case of vocabulary learning, the passive viewing of the assembly of objects on a computer screen was actually advantageous to letting children assemble the objects themselves in VR. Both the design of the learning task and the way learning was measured may have contributed to this result. However, more important than the particular mode of learning was the children’s ability to sustain attention to repetitive and boring tasks. It is not possible to directly draw the conclusion that children who struggle with sustained attention benefit more from VR than PC from our study, but at least the difference between the learning conditions was not significant for these children in contrast to their peers with high sustained attention (albeit marginally). As a lot of children struggle with concentrating on schoolwork and learning in the busy reality of the school environment, we believe it is a worthwhile aim to continue studying how we can help these children find ways of learning that motivates them. If VR could prove to be one such way for these children to find more joy in learning and to keep coming back to learning environments, it may be an important tool to reach that goal. However, before implementing VR broadly in the school setting, we need to better understand which factors contribute to learning in virtual reality, both in terms of aspects of the learning environment, the learning task, the types of knowledge we aim to teach, and individual differences in learners’ cognitive and behavioural style.

## Methods

### Participants

School aged children from Swedish middle school, years four and six were recruited from two separate schools (School A, *n* = 40; School B, *n* = 35). The inclusion criteria specified an age range between 9:0 and 13:12 years (corresponding to middle school in Sweden). One participant was excluded due to being of an age that was above the inclusion criteria (169 months, i.e., over 14 years old; the second highest age was 157 months). Another participant only participated in the cognitive testing and not the word learning or recall tests due to illness during data collection and was thus excluded from the analyses. The final sample consisted of 73 participants (38 boys and 35 girls) with an average age of 11:6 years (School A, 10.6 years; School B, 12.7 years). Recruitment was conducted through contact with the schools, which sent out written information about the study to parents and their children. Written consent were obtained from parents before participation. This study was approved by the Swedish Ethical Review Authority (Approval Number: 2023-06353-01) and was conducted in accordance with the Declaration of Helsinki.

### Procedure

The participants trained on a total of 108 words from the ancient farming equipment paradigm, during a span of two weeks, using both VR and PC in a cross-over design with the order of the learning conditions counterbalanced. For each learning condition (VR and PC) there were three sets of 18 words to train on, resulting in a total of six sets of words. In the first week, the children trained with sets 1–3 in one of the two conditions (i.e., either using PC or VR) and in the second week they trained with sets 4–6 in the other condition, independent of the order in which they trained with the two learning conditions (VR or PC). Most children trained with all 6 sets, although 8 children missed one or more training sessions. The training was performed in the schools during regular school days with a test-leader present for each training session. The children were instructed to try to learn the words to the best of their ability and that they would be tested on them afterwards. At the end of each week there was a computerized recognition test of the words from the three sets trained on that week.

Both the VR and PC training programs were developed as part of this study. Using Blender (Stichting Blender Foundation, 2018), the drawings from the ancient farming equipment paradigm were converted into 3D objects, which were then segmented into parts before being placed as interactive objects in a virtual environment resembling an old cottage (for the VR condition). Before the training started, the children participated in computerized testing of language aptitude (Llama B and D^48,49^) and sustained attention (Sustained Attention to Response Task, SART^35^), see below for descriptions. These tests were performed on laptops using headphones in the schools, supervised by a test-leader. The Llama tests were performed online (www.llamatests.org) and the SART test was performed in Psychopy (Version 2023.2.3; www.psychopy.org).

### The VR learning condition

The VR training was done individually on head-mounted Meta Quest Pro headsets and the accompanying hand controllers. Both the headset and the controllers rely on inside-out movement tracking by integrated cameras. The VR training game was implemented in the Unity 3D engine (version 2021.3.16 f, https://unity.com/) with the Oculus XR Plugin (version 3.2.3, https://docs.unity3d.com/Packages/com.unity.xr.oculus@3.2) and XR Interaction Toolkit (version 2.5.2, https://docs.unity3d.com/Packages/com.unity.xr.interaction.toolkit@2.5). The compiled application was installed and executed locally on the headsets, and logs saved as text files to the headsets’ internal storage.

**Fig. 2:**
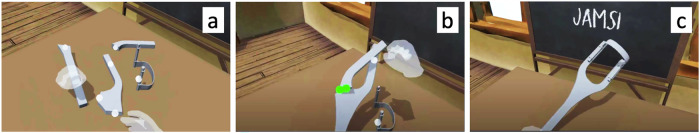
The VR condition learning task. Panels (**a**–**c**) illustrate the assembly process and observation phase in the virtual environment. **a** Four parts of an object laying on the table as viewed by the player before assembling. **b** The object as it is being assembled. The white nodes turn green and it is possible to let go of the object piece as it snaps into place. **c** The object has been correctly assembled, and the name of the object appears on the black board next to the table, as a recording of the name of the object is played three times.

In the VR training game participants were assembling objects in a virtual environment. The environment consisted of a small room with a table in front of the player and a blackboard next to it. The hand controllers were virtually represented by semi-transparent white hands, with fingers animated to open or close depending on if the ‘grip’ button on the inside of the controller (opposite the palm) was pressed. The participants’ movements were tracked with six degrees of freedom, i.e., both the positions and orientation of the viewport and hand controls were updated according to their movements. During the first phase, the assembly phase, the task presented to the participants was to put together objects which had been divided into three to five pieces (Fig. 2a). The participants used hand controls (one in each hand) to pick up pieces and put them together at specific nodes, which signalled that the pieces had been put together by turning from white to green (Fig. 2b). The interaction scheme for assembling the objects required a custom design and implementation, specifically for this study: Pieces were picked up by pressing the grip buttons; connecting or disconnecting pieces required participants to hold one piece in each hand; picking up a piece already connected to other pieces would keep them connected and lift all the connected pieces. If any piece fell to the virtual floor or participants used a specific button combination the objects were reset, i.e., all disconnected pieces reappeared on the table.

When an object had been correctly assembled, a short acoustic signal was played, and 2 seconds after the start of the signal the name of the object was presented on the blackboard next to the table for a total of 10 s (Fig. 2c), while a spoken recording of the word was played three consecutive times. During this *observation phase*, the assembled object was locked to the participant’s hand, meaning it was not possible for them to throw it or dismantle it. After the observation phase, the word was erased from the black board and a new set of pieces to assemble appeared on the table in front of the player. At the beginning of each training set there was a practice object (a kazoo). On average, participants spent 19.40 (SD = 9.57) minutes to finish each set of 18 words.

### The PC learning condition

**Fig. 3:**
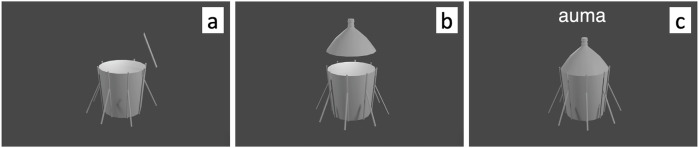
The PC condition learning task. Panels (**a**–**c**) shows the passive assembly viewing process in the PC condition. **a**, **b** The object as it is being assembled without any manual action. Parts of the objects appear one by one at the top of the screen, and fall downwards until they attach at the correct place on the main object. **c** The object has been correctly assembled, and the name of the object appears on the screen above the object as a recording of the name of the object is played three times.

The PC learning condition was developed using PsychoPy (Version 2023.2.3; www.psychopy.org). The PC training was done individually on laptops with headphones. On the screen, 15-second videos of the objects being assembled, from the same parts as the VR training, were presented without any visible hands (Fig. 3a, b). In the videos, the parts moved downwards one by one until the full object was assembled and then the resulting object rotated in the middle of the screen. Following the assembly, in the observation phase, the name of the object appeared above a still image of the object (Fig. 3c), and a spoken recording of the word was played three times for a total of 10 seconds. This set-up was chosen to make the VR and PC conditions resemble each other in that both conditions involve parts of objects being assembled, but importantly, in the PC condition this assembly was made without human, manual action. After the observation phase, participants pressed the space bar to move on to the next object in the set. It was not possible to skip any parts of the training. At the beginning of each training set there was a practice object (a kazoo). Each training session with 18 objects took 8 min.

### The recall test

**Fig. 4:**
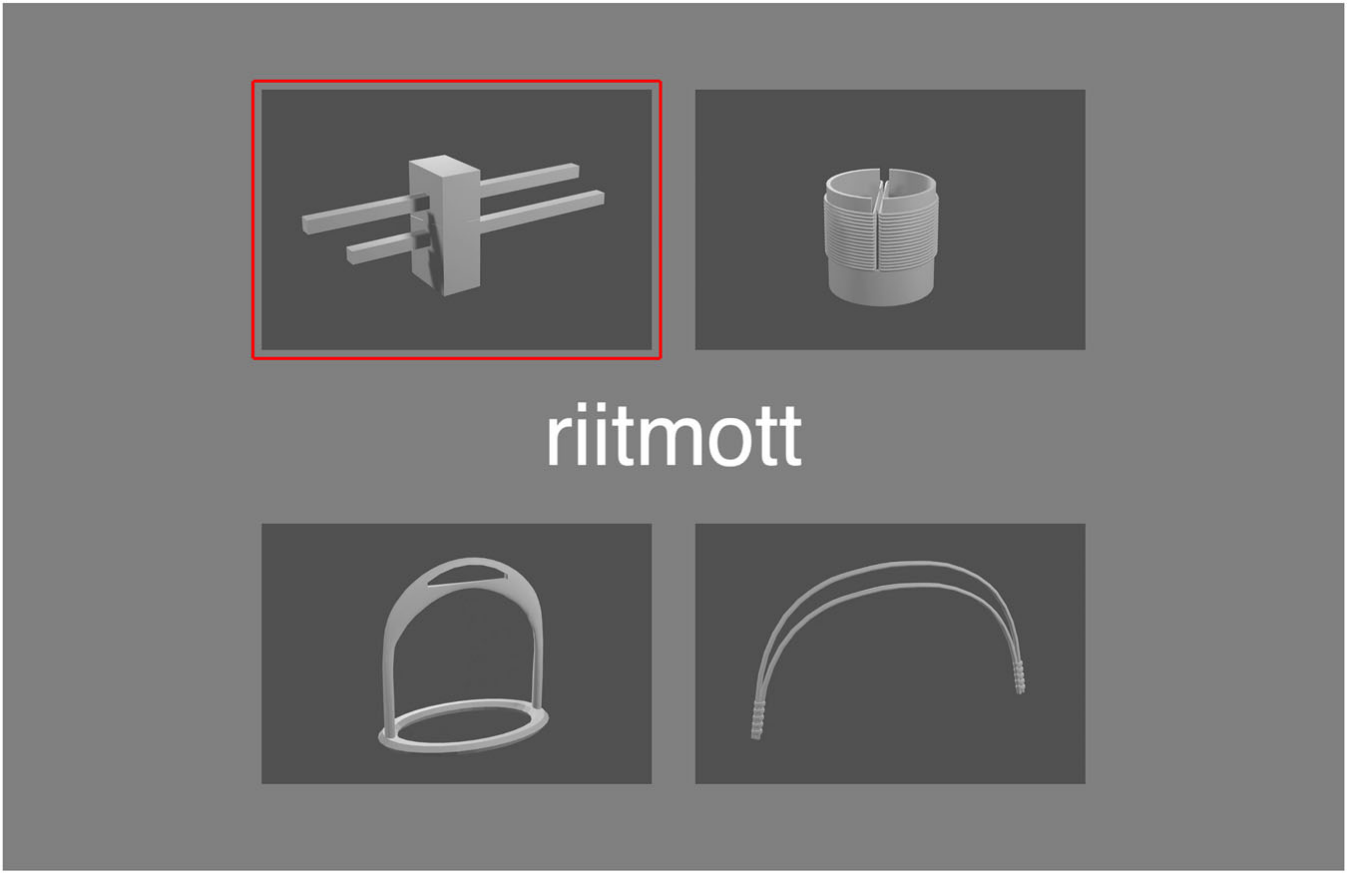
The recall test. The name of the object is displayed in the middle, and four images of objects are displayed around it. The red box indicates which image has been chosen as a match for the displayed word.

At the end of each week of training with one of the learning conditions, participants performed a recall test of the 54 object names from that week’s three sets (A-C or D-F) independent of the learning condition. The recall test was done on a separate day from the last training session. The test was done individually on laptops with headphones using PsychoPy (Version 2023.2.3; www.psychopy.org). For each object name, the written word was displayed in the middle of the screen as a spoken recording of the word was played three times. Four images of different objects from the training set were displayed around the written object name, one of which was the correct one (Fig. 4). Participants were instructed to click on the object image that they thought matched the word, and then the test moved on to the next object word. It was not necessary to listen to the whole recording before moving on to the next word. The average time spent on these recall tests was 4.47 min (SD = 1.11). At the beginning of each recall test there was a practice object (a kazoo).

### The recall score measure

The main outcome measure was the recall scores, which reflected the number of correctly identified words in the recognition tests. Each participant performed the recognition tests twice (once for each learning condition; VR and PC) and during each recognition test the participants were tested on words from the three blocks of words they had practiced on that week (blocks A-C or D-F). Due to missed school days for some of the children, not all participants completed the full set of training sessions, and in total, 6 children missed between 1 and 2 of the training sessions. To counteract these participants being tested on words that would be unknown to them, we calculated the individual average score by dividing the number of correctly identified words by the number of training blocks completed, for each participant separately. The maximum score of the Recall score is thus 18, as is the number of words in each block.

### SART (Sustained Attention to Response Task)

The Sustained Attention to Response Task (SART^35^) is a test of sustained attention failures. The task is a go/no-go paradigm, in which a sequence of digits (0-9) is shown on the screen in random order for 1150 ms, and the participant is tasked with pressing the space bar as fast as possible for each digit (go trials) except for when a ‘3’ is shown (no-go trials). Each digit is shown 25 times in a random order for a total of 225 trials. Because to the no-go stimuli (the ‘3’) is rare in relation to the large number of trials, the task is demanding on the ability to inhibit automatic responses and sustain attention to a boring, repetitive task. The measure used in the current study is the accuracy of the no-go trials, meaning the percentage of the no-go trials during which the space bar was accurately *not* pressed. The maximum possible score is 100.

### LLAMA (Language Learning Aptitude and Motivation)

The Language Learning Aptitude and Motivation test (LLAMA^48–50^) is commonly used to measure language aptitude independent of the language(s) participants are fluent in. It is computerized and the score is automatically calculated. In this study two subtests from LLAMA version 3 were used (www.lognostics.co.uk). The LLAMA B subtest assesses associative learning of new words. During the learning phase the screen simultaneously shows 20 drawings of novel creatures. When the mouse is hovered over each drawing the name of the creature is shown in writing. Participants have two minutes to practice the word-object pairings, after which the test phase begins. During the test phase, the same 20 drawings are shown, and underneath the drawings there is written an instruction to select the image that corresponds to each of the words learned. The outcome measure is the number of correct word-object pairings identified, with a maximum of 20 points. The LLAMA D subtest assesses auditory implicit word learning. Participants listen to non-words and click on one of two buttons depending on whether they think the word is novel or has been played before. There are a total of 50 items, of which the first 10 are exposure items and the following 40 are test items, resulting in a maximum of 40 points.

### VR behavioural measures

**Fig. 5:**
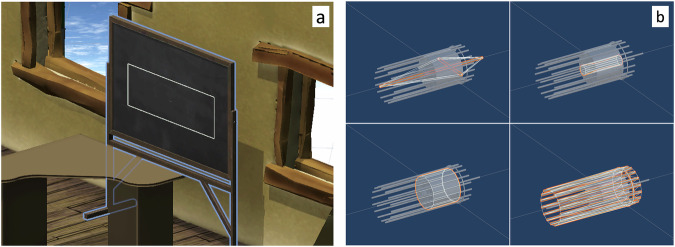
The areas of interests (AOIs) of the eye tracking analysis. **a** The Black board AOI superimposed in light blue outlines over the virtual black board. During the phase when the word is shown, it is visible within the white box. **b** Object AOIs superimposed in orange outlines over each piece of an object (here four).

The Meta quest pro VR headsets use infrared eye tracking to estimate the gaze direction of the user. This enabled us to analyse the gaze of each participant when they were training in the VR environment. The gaze vectors were extracted with a sample rate of 10 samples/second. Areas of interest (AOIs) were placed over the virtual black board (where the written word name of each object was displayed after the assembling) and over each of the object pieces Fig. 5).

To measure how much time each participant spent looking at the written word, we first extracted the 12 s period of the observation phase, i.e. from the time point the objects was assembled, to the time point when the word disappeared from the virtual black board. The measure *Word gaze* was calculated as the percentage out of the observation phase in which the participant’s gaze was on the written word and not the object. This was done by first marking the samples in which the gaze was within the black board AOI and not within any of the object AOIs. We filtered out all instances where the gaze was within the black board AOI for less than two consecutive samples (i.e., less than 120 ms^51^). We then calculated the percentage for each word/object and each participant and then aggregated this within participants.

We extracted data on two additional measures from the VR headsets: Assembling time and Rotation. Assembling time was calculated as the duration of the assembly phase, i.e., the time from when the pieces appeared on the table until the object was correctly assembled. If reset, the timer for the assembling time would also reset. Rotation was calculated as the accumulated, frame-by-frame angular rotation of the object during the observation phase.

### Control measures

Measures were collected through parental questionnaire to be used as potential control variables in the analyses. These were: *Age*, measured in full months rounded down; *Socio economic status (SES)*, determined by the average maximum educational level of the parents on a four-level scale (elementary school (1), high school (2), bachelor’s level (3) and master’s level (4); *Gender*, categorized as boy, girl, or other (no participant reported the last category); *Multilingualism*, based on the response to the question “Do you speak any other languages than Swedish at home?”; *School*, indicating which of the two included schools each participant was recruited from; *Frequency of computer games* and *Frequency of VR*, both rated by the parents on a six-point scale from “never” to “often”.

### Analyses

Analyses were performed using Jamovi version 2.6.2.0 (www.jamovi.org) and IBM SPSS version 29.0.0.0. Significance tests were two-tailed, with an alpha value of 0.05. The data was checked for normality using skewness and kurtosis measures and graphical visualisation of distributions (qq-plots and histograms). The variable *Age* had a bimodal distribution, as all participants were recruited from either school year 4 (mean age of 128 months, SD = 3.5) or 6 (mean age 152 of months, SD = 3.3). Although none of the other measures indicated substantial deviation from normality (with skewness values of < +/−2 and kurtosis values of <+/−7; Table 1; West et al.,^52^), we used bootstrapped confidence intervals and p-values when applicable (bias-corrected and accelerated (BCa) bootstrapping with 1000 samples). Further, for the Word gaze measure, which was the measure with furthest deviations from normality, we performed both parametric and non-parametric analyses (Spearman’s rho and Mann-Whitney U-test, respectively). The results from the non-parametric analyses where in line with the parametric ones.

## Supplementary information

Supplementary revision.

## Data Availability

The datasets generated and analysed in this study contain personal data and are therefore subject to GDPR requirements. Data are available from the corresponding author upon reasonable request and under a data-sharing agreement that ensures compliance with GDPR. Summary data supporting the findings are provided in the published article and its supplementary information.

## Code availability

The code used to develop the VR learning environment, the PC learning environment, and the recall test, as well as scripts for extracting and processing VR behavioural data, may be obtained from the corresponding author upon reasonable request. The VR application was implemented in Unity 3D (version 2021.3.16 f) using the Oculus XR Plugin (version 3.2.3) and XR Interaction Toolkit (version 2.5.2). The PC training and recall test were developed in PsychoPy (version 2023.2.3).
