# Supplementary material for: Embodied word learning in schools and sustained attention in virtual reality
Source: NPJ Sci Learn. 2026 Jan 10;11:9. doi: 10.1038/s41539-025-00395-2 (PMC12804862; doi:10.1038/s41539-025-00395-2)
Supplement: Supplementary file 1 — Supplementary revision. [file 41539_2025_395_MOESM1_ESM.pdf]

Embodied Word Learning in Schools and Sustained Attention in Virtual Reality

Supplementary Information

Supplementary Table 1: Correlation between control variables and the VR and PC recall scores

		PC Recall	VR Recall
**Gender (Boy/Girl)[a]**	Pearson's r	0.20	0.12
	p-value	0.094	0.339
	95% CI Upper	0.41	0.34
	95% CI Lower	-0.03	-0.12
	N	72	71
**Age (months)**	Pearson's r	0.11	0.05
	p-value	0.343	0.655
	95% CI Upper	0.34	0.28
	95% CI Lower	-0.12	-0.18
	N	72	71
**SES (parents' highest education)**	Pearson's r	0.05	-0.09
	p-value	0.695	0.458
	95% CI Upper	0.28	0.15
	95% CI Lower	-0.19	-0.32
	N	71	70
**Frequency of playing computer games**	Pearson's r	0.01	-0.06
	p-value	0.935	0.630
	95% CI Upper	0.24	0.18
	95% CI Lower	-0.22	-0.29
	N	71	70

**Frequency of playing VR games**	Pearson's r	-0.16	-0.04
	p-value	0.189	0.762
	95% CI Upper	0.08	0.20
	95% CI Lower	-0.37	-0.27
	N	72	71
**Multilingual home (Yes/No)[a]**	Pearson's r	0.15	0.02
	p-value	0.197	0.898
	95% CI Upper	0.37	0.25
	95% CI Lower	-0.08	-0.22
	N	72	71
**School A or B[a]**	Pearson's r	-0.17	-0.10
	p-value	0.159	0.406
	95% CI Upper	0.07	0.14
	95% CI Lower	-0.38	-0.33
	N	72	71

Note. * p < .05, ** p < .01, *** p < .001

[a] *Dichotomous variables, point-biserial correlation*
